# Cranial Neuropathy As Initial Manifestation of Primary Sjögren’s Syndrome: A Case Series With Literature Review

**DOI:** 10.7759/cureus.53063

**Published:** 2024-01-27

**Authors:** Hamza Lagtarna, Yahya Naji, Nawal Adali

**Affiliations:** 1 Neurology Department, Agadir University Hospital, Agadir, MAR; 2 Neurology Department, REGNE Research Laboratory, Faculty of Medicine and Pharmacy, Ibn Zohr University, Agadir, MAR

**Keywords:** primary sjögren's syndrome, acr-eular criteria, therapeutic management, immunosuppression, cranial neuropathy

## Abstract

The clinical spectrum of primary Sjögren's syndrome (PSS) extends beyond its classical manifestations. This work explores an unusual aspect of PSS, namely the initial presentation of cranial neuropathy. The study was conducted over a period of 22 months, from January 2022 to October 2023. Of 58 PSS patients, only five (four women and one man) had cranial neuropathy as their initial manifestation. Only one patient had sixth cranial nerve involvement, three had acute optic neuritis (second cranial nerve), and three had fifth cranial nerve involvement. The diagnosis of PSS was retained according to the 2016 ACR-EULAR criteria. All patients received symptomatic and immunosuppressive treatments. The course was favorable for all patients. The purpose of this case series is to show that cranial neuropathy can be the initial manifestation of PSS, which should be systematically investigated after the elimination of the most common etiologies of cranial neuropathy, particularly in the elderly.

## Introduction

Sjögren's syndrome (SS) is a slowly progressive autoimmune disease characterized by lymphocytic infiltration of the exocrine glands and significant loss of secretory function, along with dry eye and dry mouth syndrome [1]. SS is said to be primary (PSS) when it occurs in the absence of another autoimmune disease and called secondary when it is associated with an autoimmune disease such as rheumatoid arthritis, systemic lupus erythematosus, or scleroderma [2]. PSS mainly affects middle-aged women, generally between the fourth and sixth decades of life [3]. The prevalence of PSS is highly variable, ranging from 1/100 to 1/1000 [4]. The diagnosis of PSS is challenging because it is based on a combination of clinical (dry eye and mouth, Schirmer's test), biological (specific antibodies), and histopathological findings (biopsy of accessory salivary glands). PSS could have extra glandular manifestations involving many systems, including neurological disorders, observed in 10% to 60% of cases [5,6]. These manifestations may be central or peripheral, with a clear predominance of peripheral manifestations [3]. Cranial neuropathy is an uncommon symptom of PSS; any cranial nerve from the second (II) to the twelve pairs (XII) could be affected, although trigeminal sensory neuropathy remains the most common [7]. This study aims to describe cases in which PSS was first revealed by cranial nerve involvement and publish literature to remind clinicians to consider PSS in the face of cranial neuropathies.

## Case presentation

Our retrospective study covered the period from January 2022 to October 2023; it took place at the Department of Neurology at Agadir University Hospital. We reviewed the medical records of all patients diagnosed with PSS disease. From 58 patients with PSS, we found only five patients with cranial nerve lesions as initial symptoms of PSS. The average age was 50 years; four were female patients versus one male. In all patients, the diagnosis of PSS was initially revealed by cranial neuropathies and retained according to the 2016 ACR-EULAR criteria. The principal features of the cohort are resumed in Table 1.

**Table 1:** Clinical and paraclinical features of our cohort

	Case 1	Case 2	Case 3	Case 4	Case 5
Gender/age	M/49	F/49	F/61	F/74	F/36
Dry eye	+	+	-	+	+
Dry mouth	+	+	+	+	+
Acute vision loss (II)	+	+	+	-	-
Diplopia	+ (CNVI palsy)	-	-	-	-
Facial neuralgia (V)	-	+	-	+	+
Schirmer's test	5 mm	4 mm	3 mm	4 mm	5 mm
Chronic sialadenitis	Stage 3	Stage 3	Stage 3	Stage 3	Stage 4
MRI (cerebral /spinal)	Right optic nerve hyperintensities	Hyperintensities lesions in the periventricular and spinal cord	Normal	Normal	Normal
Treatment	Methylprednisolone cyclophosphamide	Methylprednisolone cyclophosphamide	Cyclophosphamide azathioprine	Azathioprine pregabalin	Hydroxychloroquine duloxetine

Case 1

**Figure 1:**
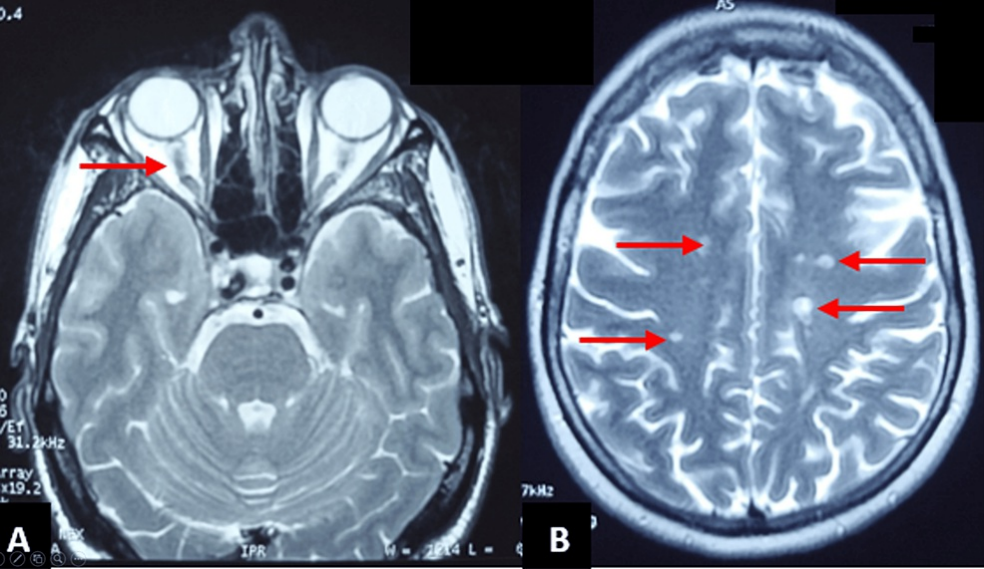
Brain and optic nerve abnormalities found in the first case admitted for acute vision loss associated with horizontal diplopia (A) Orbital MRI showing tortuosity in the optic nerves with bilateral optic neuritis (T2 sequence). (B) Subcortical white matter lesions in brain MRI (T2 sequence).

A 49-year-old man was admitted for acute vision loss and horizontal binocular diplopia. Neurological examination showed isolated involvement of the second (II) and sixth cranial nerves (VI). Ophthalmological examination revealed reduced bilateral visual acuity with manifest ocular dryness. Orbital and brain MRI showed bilateral optic nerve sheaths and subcortical white matter T2 and FLAIR hyperintensities (Figure 1). An accessory salivary gland biopsy (ASGB) revealed chronic sialadenitis (stage III). Cerebrospinal fluid (CSF) studies and blood tests were normal. The patient was treated with intravenous corticosteroid (methylprednisolone 1 g/d for three days), followed by oral prednisolone relay (60 mg/d for one month), and immunosuppressive treatment (bolus of cyclophosphamide 1 g/month for six months), along with symptomatic treatment (lubricating eye drops) and an orthoptic treatment of diplopia. The follow-up showed improvement in visual acuity with a regression of diplopia.

Case 2

**Figure 2:**
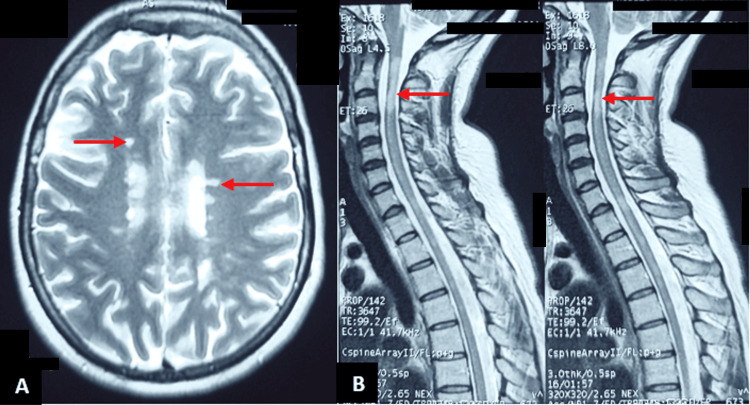
Brain and spinal abnormalities found in the second case presenting with rapidly progressive bilateral visual acuity deficit (VAD) associated with left hemifacial neuralgia (A) Brain MRI showing periventricular hyperintensities on T2. (B) Spinal cord MRI showing both longitudinally extensive spinal cord lesions over three vertebral segments and segmented ones.

A 49-year-old woman presented with tetraparesis, bilateral visual acuity deficit (VAD), and left hemifacial neuralgia. The neurological assessment showed ocular dryness and reduced visual acuity, rated at 20/30 on the right and 20/40 on the left, along with tetrapyramidal syndrome. Cerebrospinal MRI showed T2 and T2 FLAIR hyperintensities in the periventricular and spinal cord (Figure 2). The CSF study showed intrathecal IgG synthesis. ASGB showed chronic sialadenitis (stage III). The patient received symptomatic treatment (lubricating eye drops), corticosteroid (methylprednisolone 1 g/d for three days), followed by oral prednisolone relay (60 mg/d for one month), and immunosuppressive treatment (bolus of cyclophosphamide 1 g/month for six months). The evolution was marked by the improvement of hemifacial neuralgia, myelitis symptoms, and visual acuity (20/25 in both eyes).

Case 3

**Figure 3:**
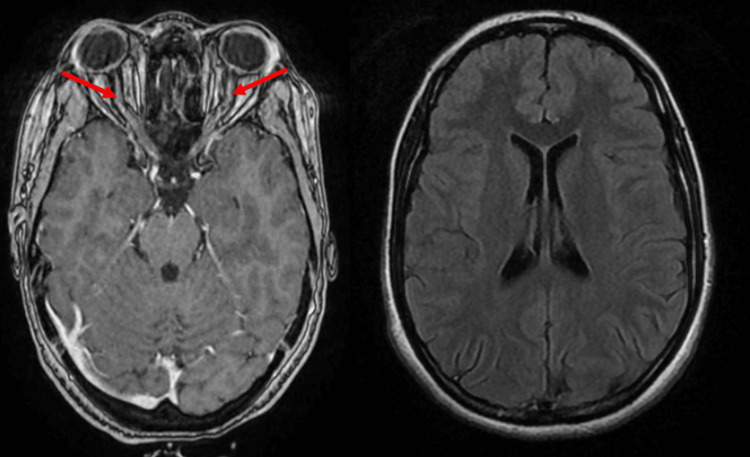
Cerebral MRI showing bilateral optic neuritis with gadolinium enhancement

A 61-year-old woman was admitted to the emergency department with acute vision loss in both eyes. Neurological examination revealed low visual acuity, rated at 20/50 in both eyes. Cerebral MRI showed hyperintensity lesions in both optic nerves (Figure 3). A CSF study showed hyperproteinorrachia with intrathecal IgG synthesis. Immunological tests were negative (ANA, anti-SSa, and anti-SSb). ASGB was in favor of chronic sialadenitis (stage III). The patient received corticosteroid (methylprednisolone 1 g/d for three days), followed by oral prednisolone relay (60 mg/d for one month), and immunosuppressive treatment (bolus of cyclophosphamide 1 g/month for six months). Along with symptomatic treatment (lubricating eye drops), The follow-up was marked by partial improvement of the visual acuity (20/25 in both eyes).

Case 4

**Figure 4:**
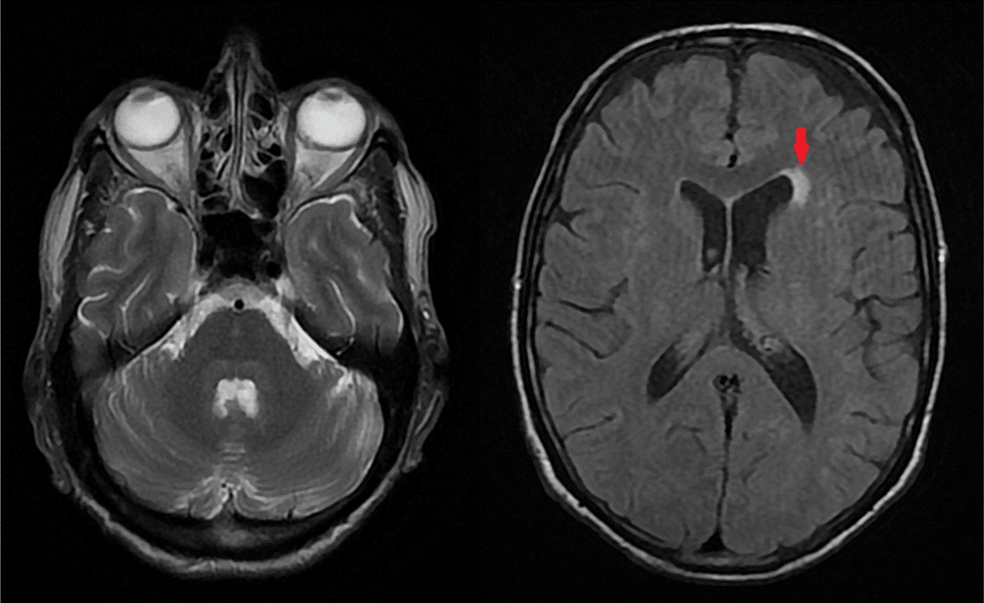
Cerebral MRI showing some vascular hyperintensities with no lesion in the pons

A 74-year-old female patient suffered from right hemifacial neuralgia. Neurological examination revealed tactile hypoesthesia in both lower limbs. Ophthalmological examination revealed significant ocular dryness. Cerebral MRI was normal (Figure 4). Electroneuromyography was consistent with multiple mononeuropathies in the lower limbs. ASGB was in favor of chronic sialadenitis (stage III). The patient was treated with azathioprine (100 mg/day) and symptomatic treatment (lubricating eye drops and pregabalin 150 mg/day). Clinical evolution was favorable.

Case 5

**Figure 5:**
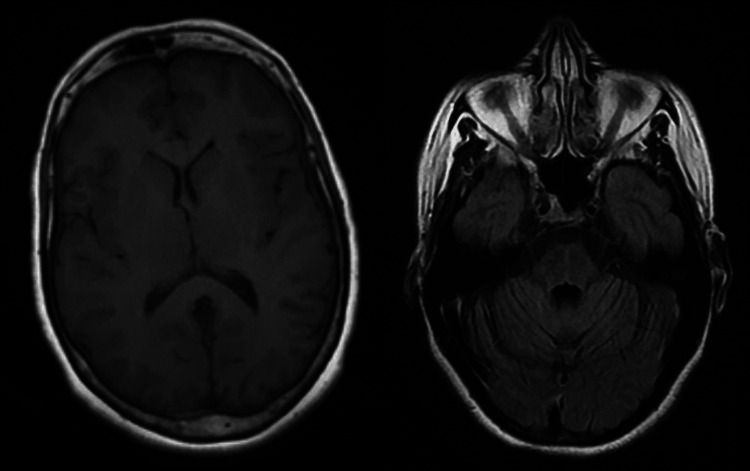
Cerebral MRI with no abnormalities

The last case was about a 36-year-old woman with a history of right hemifacial neuralgia. Neurological examination revealed hypoesthesia of the V1 and V2 territories bilaterally, while ophthalmological examination was consistent with ocular dryness. A cerebral MRI with sections over the brainstem and trigeminal nerves was without abnormalities (Figure 5). The immunological tests were positive (ANA, anti-SSA, and anti-SSB positive), and ASGB showed stage 4 chronic sialadenitis. The patient received hydroxychloroquine along with symptomatic treatment (lubricating eye drops and duloxetine). The clinical outcome was good.

## Discussion

SS is a chronic autoimmune disease in which mononuclear lymphocytes infiltrate the exocrine glands and replace the glandular epithelium. This can lead to keratoconjunctivitis and xerostomia. It is considered a systemic disease due to its various extra glandular complications, including skin rashes, interstitial pneumonia, pericarditis, and renal tubular acidosis [1]. Neurological manifestations of SS are seen in 10-60% of patients with SS; the most common form is pure sensory neuropathy (40% of neurological manifestations of PSS) [2]. Rarely, cranial neuropathy can be the initial manifestation of PSS (16% trigeminal neuropathy, 5% multiple cranial neuropathies) [8], in which case the two most common types of involvement are trigeminal neuropathy and multiple cranial neuropathies, including involvement of the facial nerve and, more rarely, the oculomotor nerves [5-7].

The pathophysiology of multiple cranial neuropathies [1] is not yet well understood. Recently, two mechanisms have been proposed: a vascular origin with damage to the vasa nervorum and an immunological cause inducing lymphocytic infiltration of the nerve, especially in the second case when improvement occurs with corticosteroid therapy [2]. Aseptic meningitis is also thought to contribute to multiple cranial neuropathies [9]. In our series, three patients had trigeminal involvement and a second cranial pair, while only one patient had involvement of the sixth cranial pair, which is almost identical to the literature data. Trigeminal neuropathy is well-known to be associated with PSS and other connective diseases. In a large cohort comprising 92 patients with PSS, 15 patients had pure sensory trigeminal neuropathy, representing 17%, nine patients with unilateral involvement, and six with bilateral involvement [10]. Most trigeminal neuropathies can occur initially or appear later in patients with sensory ataxia or painful small-fiber sensory neuropathy. The pathophysiology of trigeminal sensory neuropathy is unclear. It may be related to myelin sheath loss, axonal degeneration, or vasculitis. It has also been reported that trigeminal denervation is due to lymphocytic infiltration of the trigeminal ganglion [5,10,11].

Involvement of the oculomotor nerves has been described rarely in the literature. Only 10 cases with third cranial nerve involvement and 21 cases with involvement of the abducens nerve have been reported in the literature so far [12]. In our series, three of our patients had optic neuritis. According to the literature, PSS-related optic neuritis is seen in 5.8-6.3% of reported cases of optic neuritis; it can be unilateral or bilateral, progressing rapidly or insidiously to chronic optic atrophy, or evolving as part of a spectrum of neuromyelitis optica [13]. Another study described eight cases of PSS with optic neuritis; all of them were women, and four of the eight cases had bilateral optic neuritis. Similarly, in a retrospective analysis of 424 patients with PSS, around 5.8% of patients had central nervous system involvement, including optic neuropathy. Occasionally, optic neuropathy can be the initial manifestation of PSS, making the diagnosis more difficult [1,13,14].

None of our patients presented with facial nerve involvement. In a literature review on cranial neuropathy in PSS, facial paralysis was described in 23 patients. Ten cases had facial paralysis as part of multiple cranial neuropathies, while thirteen had pure facial paralysis; two of them were bilateral. In addition to PSS, facial paralysis can be a symptom of Lyme disease, Guillain-Barre syndrome, sarcoidosis, HIV infection, and central nervous system lymphoma, especially in cases of bilateral involvement [15]. Multiple cranial neuropathies are relatively rare in PSS; they should be considered in other conditions besides PSS, such as diabetes, neoplasticity, and infectious diseases [1].

The therapeutic management of PSS has not changed considerably in recent decades; it remains based on symptomatic treatment of sicca symptomatology and broad-spectrum immunosuppression for systemic disease [16]. For cranial neuropathies, the use of corticosteroids, cyclophosphamide, rituximab, and plasma exchange has been proven to be effective [5,16,17]. However, treatment decisions remain challenging in clinical practice [16].

## Conclusions

Cranial neuropathies, as an initial presentation of PSS, underline the diverse and sometimes elusive nature of this autoimmune disorder. PSS should be considered as an underlying etiology of cranial neuropathy after excluding the most common causes, especially in elderly people. Recognizing the potential involvement of cranial nerves in the early stages of the disease is crucial for good management, particularly in a multidisciplinary approach.
